# Male-Male Sexual Behavior in the Parasitic Wasp *Psyttalia concolor*

**DOI:** 10.1673/031.013.2501

**Published:** 2013-04-03

**Authors:** Giovanni Benelli, Angelo Canale

**Affiliations:** Entomology Section, Department of Agriculture, Food and Environment, University of Pisa, via del Borghetto 80, 56124, Pisa, Italy

**Keywords:** adaptive behavior, courtship, experience, mating, Opiinae, wing fanning

## Abstract

The role of male-male courtship in parasitic Hymenoptera is poorly known. A laboratory study was conducted to assess if *Psyttalia concolor* (Szépligeti) (Hymenoptera: Braconidae) male courtship can be affected by a previous experience in courting young conspecifics of both sexes. Two experiments were performed to evaluate the effect of experience in courting young wasps on both male courtship and male-male competition behavior. Results showed that a courting experience on both sexes can modify some sexual traits in a *P. concolor* male, without affecting its success in mating. When approaching virgin females, a *P. concolor* male that had a previous courtship experience with young wasps of either sex showed shorter latency times, more wing fanning, and longer courtship durations with respect to the control male. The hypothesis that a previous courting experience may allow a *P. concolor* male to refine its courtship behavior and to enhance courtship intensity in subsequent encounters with females was discussed.

## Introduction

*Psyttalia concolor* (Szépligeti) (Hymenoptera: Braconidae) is a koinobiont larval-pupal endoparasitoid of at least fourteen tephritids on different wild and/or cultivated plants, including pests of great economic importance, such as the Mediterranean fruit fly and the olive fruit fly (Wharton 1997). *P. concolor* develops on tephritid larvae that live on a wide range of small spherical fruits, generally drupes, or small globular inflorescences such as *Capparis spinosa* (Fischer 1971; Benelli and Canale 2012a). It was originally described by Marchai (1910), and shortly thereafter it was introduced to olive-growing regions of Italy and France. In the 1950s, following the development of an efficient mass-rearing technique on *Ceratitis capitata* (Diptera: Tephritidae), augmentative releases of the species against the olive fruit fly, *Bactrocera oleae*, were started in the Mediterranean areas. The releases continue to this day with limited results (Daane and Johnson 2010). More recently, the braconid was released in Californian olive groves as part of local biological control programs (Wang et al. 2011).

Shortly after their emergence, *P. concolor* males start searching for females (Benelli et al. 2012b). *P. concolor* males start walking around, performing intense antennal drumming series (i.e., female searching). When they come into close proximity of a female, they stop walking and drumming and remain still, waving their antennae in various directions. Immediately after, they start to court the female, performing short wing fluttering bursts (i.e., wing-fanning behavior). Then, the male approaches the female with one or more mating attempts, mounting the female from the back or the side, and making repeated antennal contacts with the female's head and antennae (i.e., copulation attempt). A receptive female remains still while the male vibrates its wings, folds both pairs of wings over the abdomen, and maintains the antennae back together over the wings (i.e., acceptance position). Unreceptive females walk away from males. During copula, the female remains completely immobile, and the male continues to do antennal tapping on the female's head and thorax (Benelli et al. 2012b; Canale et al. 2012).

Interestingly, *P. concolor* males display wing fanning and copulation attempts toward other males (Benelli et al. 2012a), as already noted for other braconids, such as *Aphidius ervi* and *Diachasmimorpha longicaudata* (Sivinski and Webb 1989; Xiong 2008). However, for the two latter species, no explanations were given on the occurrence of sexual behavior among males. Recently, Benelli and Canale (2012b) showed that *P. concolor* males courted by other males while they were still young showed some differences in their courtship traits. Even if *P. concolor* immature males do not appear to gain from receiving male courtship, they develop higher courtship intensities in successive approaches to females (Benelli and Canale 2012b). The role of male-male sexual behavior has been described in many other insect species (Bailey and Zuk 2009). Although some adaptive (McRobert and Tompkins 1988; Preston-Mafham et al. 2006; Dukas 2010) and non-adaptive (van Gossum et al. 2005) explanations have been given for male-male courtship, its role remains debated.

Although some researchers have documented no effects of previous experience on sexual behavior among insects (Shuker and Day 2002), courtship behavior is influenced by developmental conditions and social experiences in many species (Dukas 2006). In species in which males provide females with food gifts (e.g., tree crickets), males rely on experience to optimally adjust gift size (Bussiere et al. 2005). In *Lasioglossum zephyrum, L. figueresi*, and *Nomia triangulifera*, males that unsuccessfully court a female show higher courtship intensities when exposed to another female, with respect to inexperienced males (Greenberg 1982; Wcislo 1992). Among parasitic wasps, a previous experience can affect courtship and mating in the pteromalid *Nasonia vitripennis* (Baeder and King 2004) and in the braconid *Aphidius ervi* (Villagra et al. 2005, 2008). Finally, for *Drosophila* spp., active male-male sexual events affect the mating performances of males (McRobert and Tompkins 1988), highlighting the intriguing hypothesis that fruit fly males could learn to refine their courtship performances in further female's approaches (Dukas 2006, 2008, 2010).

In this paper, whether *P. concolor* male mating performances are affected by courting experiences on young conspecifics is discussed. We hypothesize that an active sexual experience toward other males or females could modify the *P. concolor* male courtship traits. Therefore, the courtship performances of (a) male-trained males, (b) female-trained males, and (c) inexperienced males were evaluated toward both sexes. In addition, allowing two males to compete for a female, the courtship performances of (d) males trained on young males, (e) males trained on young females, and (f) inexperienced males (controls) were put to the test.

## Methods and Materials

### Parasitoid and host rearing

*P. concolor* and its host *C. capitata* were reared in Pisa (Italy) as described by Benelli and Canale (2012a) and Canale and Benelli (2012). To obtain virgin *P. concolor*, emergent males and females were sexed and stored singly in glass vials (diameter 10 mm, height 60 mm) at 21 ± 1° C, 48 ± 10% RH, and 16:8 L:D photoperiod, and fed with a semisolid diet (honey mixed with pollen) and water.

### General observations

Experiments were conducted in a 12 m^2^ room illuminated with daylight fluorescent tubes. These tubes were placed in such a way as to guarantee that the intensity of light was as even as possible. The temperature was set at 22 ± 1° C and the relative humidity at 45 ± 5%. Bioassays were all performed during May and June 2011, between 09:00 and 17:00. Each wasp was used only once. Both in Experiments 1 and 2, before the training phase began, males to be trained on young conspecifics were marked with a small dot of atoxic color paint (Polycolor 256, www.maimeri.it) on the thorax (Dukas and Mooers 2003). Preliminary assays revealed that this treatment did not influence subsequent behavior of the wasps (Benelli and Canale 2012b).

### Experiment 1: effect of experience in courting young wasps on male courtship behavior

**Purpose.** The aim of this experiment was to assess if *P. concolor* male courtship traits can be affected by a previous experience in courting young conspecifics of both sexes.

**Training.** A two-day-old virgin male was placed in a rectangular glass arena (50 mm large, 45 mm high, and 50 mm wide) with two young (3–10 hours old) virgin males or females, and it was observed for a 15-min training phase, in which the male courted. Control males (two days old) were inexperienced (i.e., the training phase was replaced with 15 min in an empty arena).

**Treatments.** After training, three treatments were performed: (i) one control male was tested with two virgin males or females (two days old); (ii) one male-trained male was tested with two virgin males or females; (iii) one female-trained male was tested with two virgin females. Each trial lasted 6 min; 70 replicates were done for each treatment.

**Collected data.** For each trial, the following mating parameters were recorded: (1) if the *P. concolor* male initiated searching behavior (i.e., walking and drumming activity), (2) if the male showed a wing fanning behavior, (3) the latency time (according to Bourdais and Hance (2009), the time elapsed before wing fanning commenced, which is related to the excitation of the male), (4) the courtship duration (i.e., the time that the male spent following the female, performing fanning and copulation attempts), (5) the male success in mating (i.e., if the courting male achieved a successful mating), and (6) the copula duration.

**Data analysis.** Data were processed by JMP 7^®^ (www.jmp.com), using a weighted generalized linear model with one fixed factor: y = Xß + ε, where y is the vector of the observations (e.g., number of searching and courting males, courtship and copula duration, mating success), X is the incidence matrix, ß is the vector of fixed effects (i.e., the male training: inexperienced, male-trained, or female-trained males), and ε is the vector of the random residual effects (Sprinthall 2003).

### Experiment 2: effect of experience in courting young wasps on male-male competition

**Purpose.** The aim of this experiment was to assess if a previous experience in courting young conspecifics of both sexes has any effeet on *P. concolor* male-male competition for a female.

**Treatments.** Two treatments were performed: (i) male-trained male and an inexperienced male, or (ii) a female-trained male and an inexperienced male, were placed in the testing arena with a two-day-old virgin female. Each trial lasted 6 min; 35 trials were done for each treatment.

**Collected data.** For each trial, the following mating parameters were recorded: (1) which *P. concolor* male displayed wing fanning behavior first, (2) its relative latency time, (3) which male achieved a successful mating first, and (4) its relative copula duration.

**Data analysis.** Data were processed using the general linear model described in Experiment 1, where y is vector of the observation (e.g., number of searching and courting males, copula duration, mating success), X is the incidence matrix, ß is the vector of fixed effects (i.e., the male training: inexperienced, male-courted or male-exposed males), and ε is the vector of the random residual effects (Sprinthall 2003).

## Results

### Experiment 1

**Figure 1.**
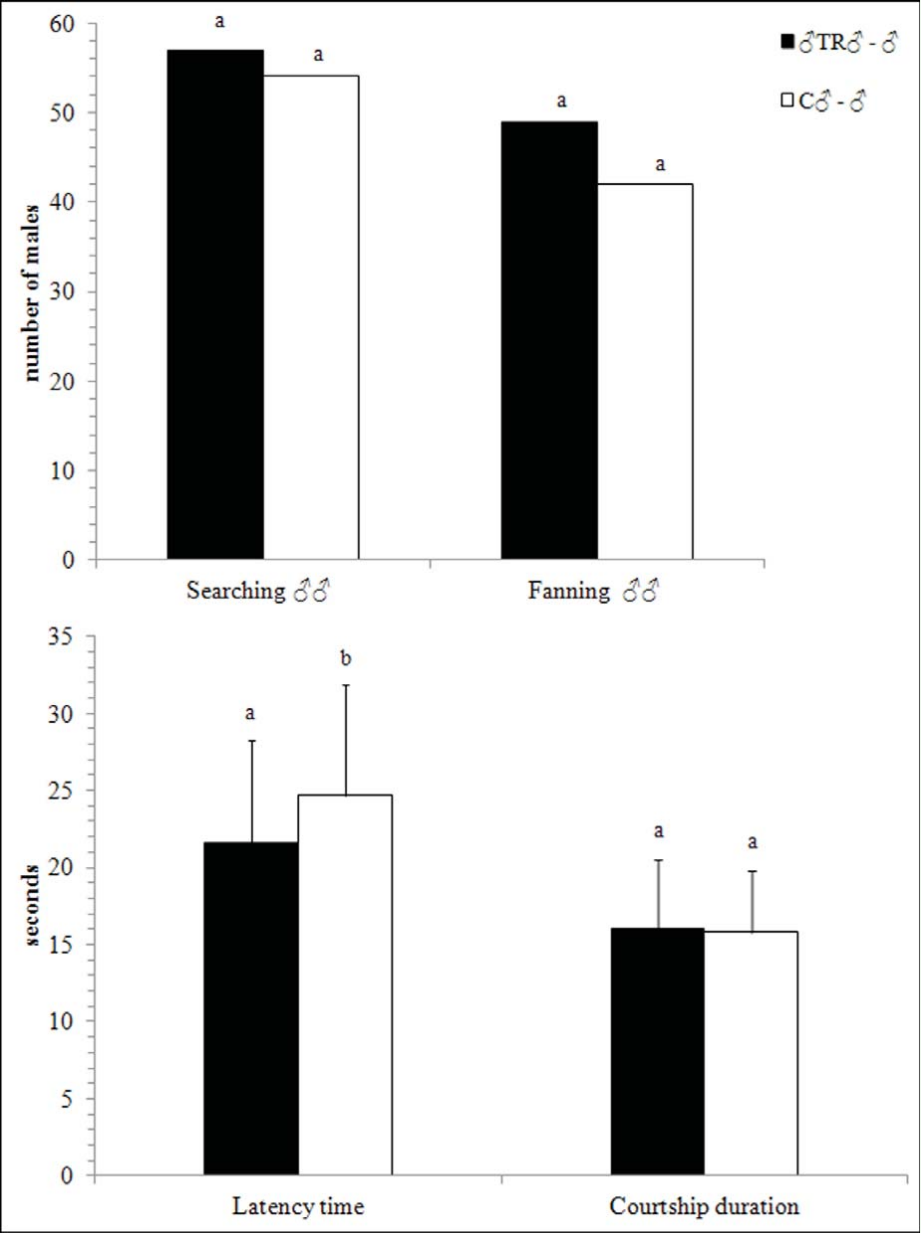
Experiment 1. Male-male courtship behavior of *Psyttalia concolor* males that had a previous experience in courting young males, with respect to inexperienced males. (♂TR♂ = males that had a previous experience in courting young males; C♂ = control males (no prior experience). Error bars represent standard deviations. For each measured parameter, different letters indicate significant differences at *p* < 0.05 (n = 70; GLM, χ^2^ post hoc test). High quality figures are available online.

The number of male-trained males that started a searching behavior or fanned their wings in the presence of another male was not different from the control (Figure 1). The latency time was shorter in male-trained males than in controls (χ^2^ = 9.152; *p* = 0.002), but they did not spend significantly more time in courting a male (Figure 1).

**Figure 2.**
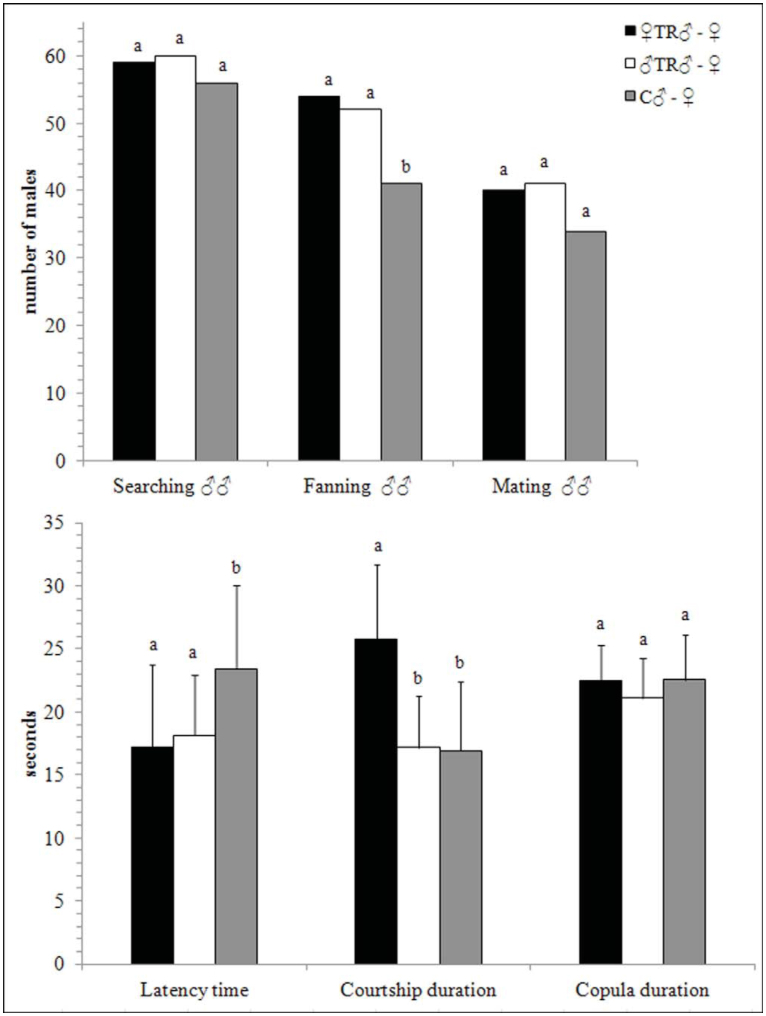
Experiment 1. Male-female courtship of *Psyttalia concolor* males that had a previous experience in courting young females or males, with respect to inexperienced males. ♀TR♂ = males that had a previous experience in courting young females; ♂TR♂ = males that had a previous experience in courting young males; C♂ = control males (no prior experience). Error bars represent standard deviations. For each measured parameter, different letters indicate significant differences at *p* < 0.05 (n = 70; GLM, χ^2^ post hoc test). High quality figures are available online.

In the presence of a female (Figure 2), the number of female-trained males that started a searching behavior was not different from the control, while males that fanned their wings (χ^2^ = 5.594, *p* = 0.018), the latency (χ^2^ = 45.336; *p* < 0.0001), and courtship durations (χ^2^ = 78.53; *p* < 0.0001) were significantly different (Figure 2). No differences were detected in copula duration and number of matings. The number of male-trained males that initiated searching behavior in the presence of a female was not different from the control (Figure 2). However, the number of male-trained males that fanned their wings (χ^2^ = 5.410, *p* = 0.020) was higher if compared to control ones. The mean latency time (χ^2^ = 32.611; *p* < 0.0001) was shorter in maletrained males, with respect to the control (Figure 2). No differences were detected in courtship and copula duration or mating success.

### Experiment 2

**Figure 3.**
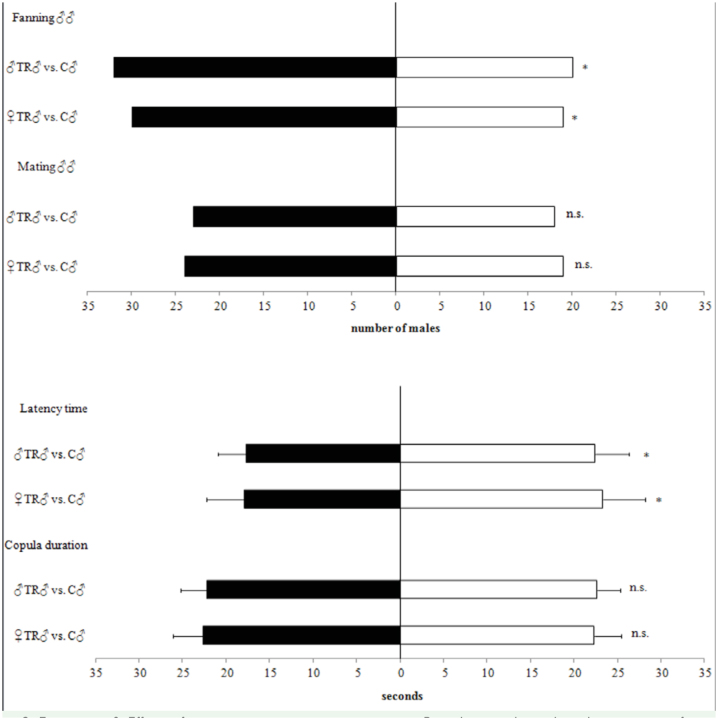
Experiment 2. Effects of experience in courting young wasp on Psyttalia concolor male-male competition for a virgin female. ♀TR♂ = males that had a previous experience in courting young females; ♂TR♂ = males that had a previous experience in courting young males; C♂ = control males (no prior experience). Error bars represent standard deviations. For each measured parameter, different letters indicate significant differences at *p* < 0.05 (n = 35; GLM, χ^2^ post hoc test). High quality figures are available online.

Allowing two males to compete for a female, the latency time was shorter both in maletrained (χ^2^ = 20.918; *p* < 0.0001) and in female-trained males (χ^2^ = 21.368; *p* < 0.0001) with respect to the control (Figure 3). The number of males that fanned their wings was higher both in male-trained and female-trained males with respect to the control (χ^2^ = 8.985, *p* = 0.0027; χ^2^ = 8.550, *p* = 0.0035, respectively). Copula duration between treatments was not significantly different, nor was the number of matings (Figure 3).

## Discussion

The results showed no differences in mating success and copula duration, both for maletrained and female-trained males, with respect to the control (experiment 1 and 2). However, shorter latency times and more fanning behaviors were recorded in both male- and femaletrained *P. concolor* males, both in courtship and in male-male competition.

Interestingly, the male-male active courting experience produced effects on courtship intensities that were similar to those observed by Benelli and Canale (2012b). They also reported that *P. concolor* males courted by other males while they were still young developed higher courtship intensities in their subsequent approaches to females. Their recent study also showed that a previous courting experience can influence *P. concolor* male sexual behavioral traits, without affecting its success in mating. Our study found no differences in mating success and copula duration between males trained on both sexes versus the control. These results are in agreement with a study by McRobert and Tompkins (1988), in which *Drosophila melanogaster* males that had courted young conspecifics showed no differences in mating success with respect to a control. On the other hand, we found that experienced males had shorter latency times than controls, whereas McRobert and Tompkins (1988) reported no effect of experience on latency *for D. melanogaster* males.

Adaptive hypotheses for the role of male-male sexual behavior have been proposed for several insect species (for a review see Bailey and Zuk 2009). In the dung fly, *Hydromyza livens*, males might reduce the mating success of competitors, while increasing their own, through same-sex interactions (Preston-Mafham et al. 2006). In *Drosophila* spp., young flies can learn mating skills through male-male courtships (McRobert and Tompkins 1988; Dukas 2010). Other studies offered non-adaptive hypotheses for same-sex copulation. In the coenagrionid *Ischnura elegans*, female-deprived males were induced to engage in same-sex activities (van Gossum et al. 2005). Both in the pteromalid *Lariophagus distinguendus* and in the tephritid *B. oleae*, no advantages were found, since it was reported that male-male courtships are due to weak sex discrimination based on olfactory ambiguity (Ruther and Steiner 2008; Benelli et al. 2013). Based on the present study, *P. concolor* functionally increased courtship intensities. *P. concolor* is a proterandrous species, and males emerge first and try to mate with females on the natal patch as soon as they emerge. Under these conditions, a previous courting experience may allow *P. concolor* males to refine their courtship performances and to perform higher courtship intensities in the successive female's approaches, as already suggested by Dukas (2005, 2010) for *D. melanogaster*. According to Vasey et al. (2008), and as extensively analyzed by Bailey and Zuk (2009) for other species, the possibility that *P. concolor* same-sex sexual interaction arises as a by-product of selection on a separate trait, such as high sexual responsiveness, cannot be excluded. Further research is required to clarify this point and to evaluate if *P. concolor* wasps that have already mated or not display different traits in same-sex sexual interactions.
